# The LGBTQI health forum: an innovative interprofessional initiative to support curriculum reform

**DOI:** 10.1080/10872981.2017.1306419

**Published:** 2017-04-12

**Authors:** Hannan M. Braun, David Ramirez, Greg J. Zahner, Eva Mae Gillis-Buck, Heather Sheriff, Marcus Ferrone

**Affiliations:** ^a^ School of Medicine, University of California, San Francisco, San Francisco, USA; ^b^ School of Nursing, University of California, San Francsico, San Francisco, USA; ^c^ Departments of Radiology & Biomedical Imaging, Clinical Pharmacy, and Family Health Care Nursing, University of California San Francisco, San Francisco, USA

**Keywords:** LGBT, disparity, syllabus, diversity, cultural competence, interprofessional education

## Abstract

Lesbian, gay, bisexual, transgender, queer, and intersex (LGBTQI) individuals continue to face barriers to accessing appropriate and comprehensive healthcare. Compounding this problem, healthcare trainees report few training opportunities and low levels of preparedness to care for LGBTQI patients. In 2009, an interprofessional group of students and a faculty advisor at the University of California, San Francisco, developed a novel student-organized LGBTQI Health Forum for medical, dental, pharmacy, nursing, and physical therapy students to deliver LGBTQI health content that was otherwise absent from the formal curriculum. This elective course has evolved based upon participant feedback, emerging educational strategies, and the existing curricula infrastructure at our institution. After eight years of growth, this 10-contact hour weekend elective attracts over 250 participants each year. Plenary sessions deliver foundational terminology and skills to all attendees. Learners then select breakout sessions to attend, allowing for an individualized curriculum based upon specific interests and knowledge gaps. Breakout session topics prioritize traditionally underrepresented aspects of LGBTQI health in professional school curricula. This Forum serves as a model in which to supplement LGBTQI content into existing school curricula and offers an opportunity for interprofessional education. Next steps include conducting a formal evaluation of the curriculum, expanding our performance-based assessments, and potentially implementing a continuing education program for licensed practitioners. With a core group of interprofessional student organizers and a faculty champion, other institutions may view this course architecture as a potential way to offer learners not only LGBTQI content, but other underrepresented subjects into their own educational programs.

## Introduction

A growing body of evidence highlights the ongoing health disparities that negatively impact lesbian, gay, bisexual, transgender, queer, intersex (LGBTQI), and gender nonconforming communities [1]. The inequities often result from gaps in providers’ clinical education and patients’ prior negative interactions, the latter characterized by the refusal of treatment, poor bedside manner, and verbal abuse from providers. In response to these inequities, the US Department of Health and Human Services published an ongoing annual report detailing efforts to combat LGBTQI health disparities and the Joint Commission issued a field guide to aid hospitals in providing more appropriate care. Numerous groups, including the American Association of Medical Colleges [2] and other professional organizations, have called for incorporation of LGBTQI health content into curricula for health professionals.

Despite these efforts, a paucity of LGBTQI-specific health knowledge and training persists among providers. Over half of transgender patients reported teaching providers about their healthcare [3], 79% of registered nurses recounted no specific LGBTQI training [4], and 65.1% of adolescent health practitioners cited lack of training as a major barrier in serving transgender youth, despite 86.4% desiring such instruction [5]. Nonetheless, students with frequent encounters with LGBTQI patients take more culturally inclusive histories, report more positive attitudes, and demonstrate greater knowledge about LGBTQI-specific health needs [6].

In practice, existing LGBTQI curricula have been underwhelming and slow to change in response to this well-characterized curricular desideration and in the face of the benefits of LGBTQI patient exposure. A recent survey of 2262 medical students found that 67.3% rated their LGBTQI curricula as fair, poor, or very poor [7]. A 2011 study revealed that US and Canadian medical schools dedicated a median of five hours to LGBTQI health in their entire curricula [8], with additional studies showing similar gaps in graduate medical training and other health professional programs. Furthermore, accreditation standards for health profession schools lack specific LGBTQI educational outcomes, although the Association of American Medical Colleges has published a guidance document to assist institutions in implementing climate and curricular change in medical education programs [2]. The glaring absence of LGBTQI-focused curricula stands in the face of a demand for such content, and there is a dearth of literature documenting successful execution.

We describe the creation and implementation of a novel student-organized, interprofessional elective to address the LGBTQI educational gap within our institution. We hope that by sharing this curricular innovation and our plans for further evaluation and improvement, other institutions may benefit.

## Design

### Setting

The University of California, San Francisco (UCSF) is comprised of four professional schools – medicine, dentistry, pharmacy, and nursing – as well as a physical therapy program administered jointly between UCSF and San Francisco State University. With guidance from faculty advisors, the university supports the development of student-designed electives, empowering students to be agents in curricular reform.

In 2006, UCSF students reported just two sessions totaling four contact hours dedicated specifically to LGBTQI health in the medical school; students from other UCSF schools reported zero hours. Therefore, students in the Lesbian, Gay, Bisexual, Transgender, Queer Student Association (LGBTQSA, a student organization within UCSF), concerned about the lack of LGBTQI course content, established a 1-unit, 10 contact hour, winter elective known as the LGBTQI Health Forum (‘the Forum’) in 2009 to deliver otherwise absent LGBTQI health content.

### Goals

**Figure 1.**
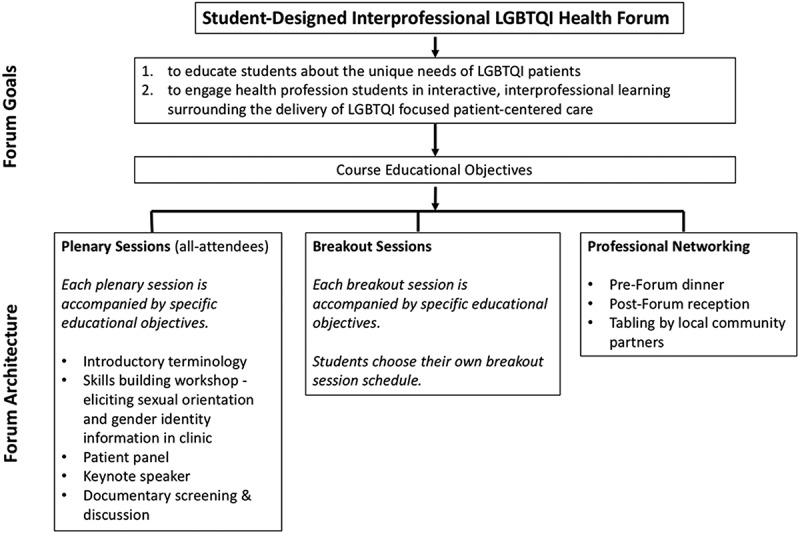
LGBTQI Health Forum goals and infrastructure. This Figure represents only the general structure of the annual Forum and a sample of content, it does not fully characterize any one particular Forum from a given academic year.

The Forum was designed at its inception to simply supply fundamental LGBTQI health content to students. Over time, the structure and function of the Forum has evolved based upon participant feedback, evolving/emerging topics in LGBTQI health and the desire to integrate into the existing institutional curriculum. Specific Forum goals are provided in Figure 1.

### Development

#### Parties and roles

A Forum planning committee organized by the LGBTQSA initiates the efforts needed to execute the elective course each academic year. We intentionally seek representation from all five professional schools for our planning committee, totaling approximately 15–20 UCSF students who meet weekly during the fall quarter preceding the elective. Representation from each school underscores the interprofessional nature of the elective even during the early design phase of the course. By placing students together at regular intervals to focus on the common goal of implementing the Forum, students embark upon some of the core principles of interprofessional practice such as understanding teams and the roles and responsibilities of team members. Members of the planning committee also engage in discussions surrounding scope of practice and professional roles in the context of providing healthcare to LGBTQI patients, as these become paramount in terms of creating the Forum schedule and its content. A balanced committee composition from all health professional schools also facilitates publicity; to illustrate this point, following a year when the planning committee lacked dental school representation, the addition of a dental student led to a doubling of dental school attendance and a new breakout session taught by dental faculty.

Subcommittees focus on fundraising, curriculum development, and publicity. Students draft grants and intra-mural funding requests, brainstorm breakout session topics, invite speakers, and coordinate announcements of the Forum on social media or traditional advertisements throughout campus. Given that planning committee membership changes as students advance in their academic programs, the execution of the Forum falls primarily on established administrative leadership working with the LGBTQSA to ensure that timelines are met and there is adherence to internal academic policies. Due to growing attendance of the Forum and the increasing workload of planning, the UCSF LGBT Resource Center now employs two student interns with protected time and financial support to serve as Forum leaders. The Forum leaders convene the planning committee and function as project managers ensuring that milestones are met and facilitating any tasks that exist on a critical path. Further guidance, oversight, continuity, and succession planning is bestowed by senior students, the faculty advisor, and the Director of the LGBT Resource Center. The faculty advisor is also responsible for securing grants to fund the course in addition to offering expertise in health profession curriculum design and evaluation. The learning management system (LMS), an online portal that supplies participants with a formal program, Forum schedule, course syllabus, updated learning materials, assessments, and additional resources, is managed by the faculty advisor who coordinates with Forum speakers and faculty to procure all learning resources and then post them in the LMS. Invited speakers work directly with the faculty advisor to construct learning objectives and assessments for their respective sessions.

#### Structure of the Forum

The Forum is comprised of three distinct components – plenary sessions, breakout sessions, and networking sessions (Figure 1). To adhere to a student-centered professional pedagogy encompassing both critical thinking and practical skills, the Forum utilizes numerous educational methodologies, including traditional didactic lectures, discussion panels, team-based learning, and performance-based assessment. The exact structure of any given plenary or breakout session is dependent upon the topic and speaker experience; however, guidance and collaboration with the faculty advisor ensures that active learning is incorporated into the session as well as a competency of interprofessional practice – such as values/ethics, roles/responsibilities, communication, or teamwork.

The Forum thrives without protected faculty time; however, participating UCSF faculty serving as speakers can document their contributions and receive student evaluations of teaching, both of which can contribute to their tenure and promotion. The annual budget of approximately US$10,000–15,000 covers modest external speaker honoraria, transportation for the keynote speaker, and at least two meals for attendees. We seek funding from both intra- and extramural grants, including private LGBTQI professional or community organizations and the Dean’s Office from each professional school at UCSF. Participants also pay a US$5–20 registration fee, which is waived for volunteers and low-income attendees.

**Figure 2.**
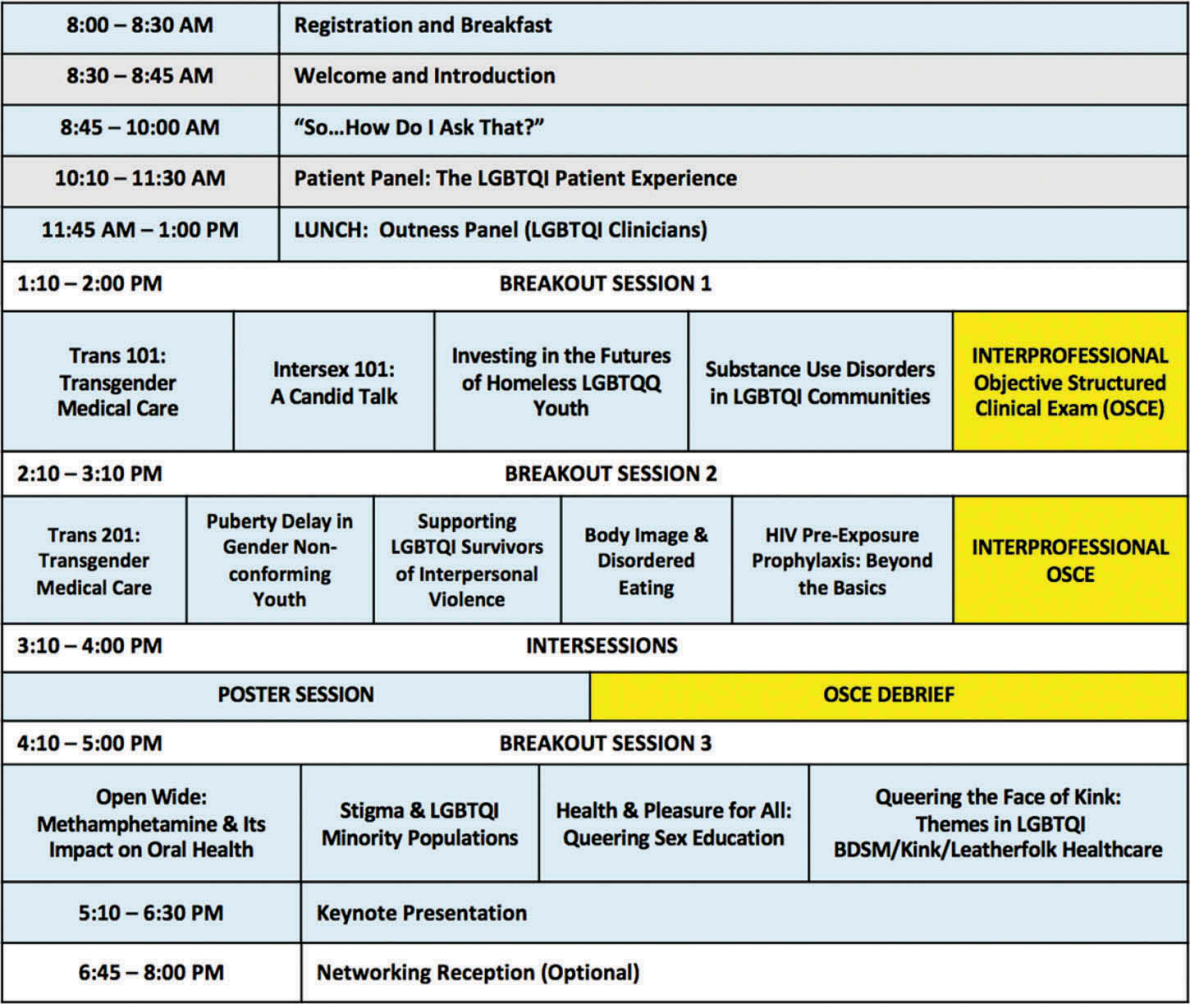
Example LGBTQI Health Forum one-day schedule. The exact Forum schedule varies from year to year, as do specific session objectives and student assessments.

The Forum occurs during winter quarter on a Saturday or as a split Friday-Saturday format on-campus. The date is established the summer before the academic year and strategically set with input from all professional schools to avoid overlap with school-specific programming. The one-day format is less expensive to execute and potentially easier for working or traveling students, although it provides fewer opportunities for informal networking and presents the challenge of engaging students for 10 consecutive hours. Figure 2 illustrates a typical one-day Forum schedule, although it should be noted that the exact itinerary varies from year to year.

The Forum commences with a plenary dedicated to LGBTQI health disparities, barriers to care, and communication skills incorporating the lexicon of LGBTQI terminology. A skills-building workshop then supplies students with the necessary patient interviewing techniques to elicit information on sexual orientation and gender identity in clinical settings.

During the patient panel, patients representing each of the LGBTQI sectors describe personal histories and experiences of substandard care resulting from provider bias, discomfort, or lack of knowledge. Our panel is further enhanced by the strategic leadership of the moderator, who initiates dialogue between the students and patients, highlighting the multidisciplinary approach for comprehensive patient care – such as the impact recreational drugs have on dental health among men who have sex with men or how a community-level pharmacist can provide LGBTQI-inclusive care. The competency domains of interprofessional collaborative practice naturally come to fruition with this open and active discussion.

Students choose breakout sessions, tailoring their educational experience within the elective, to reflect their personal and professional interests. This also allows the Forum to maintain the interest of repeat attendees. Breakout topics are based on perceived gaps in our core curricula, relevance to attendees in each of the five health professional schools, emerging topics in LGBTQI health, and availability of local experts. The existing national average of five teaching hours on sexual and gender minorities in medical school curriculum tends to focus primarily on HIV/STI diagnosis and treatment [7,8]. Therefore, our curriculum exposes students to underrepresented topics such as transgender and intersex health, mental health issues, kink health, and LGBTQI primary care. Finally, since pre-existing knowledge of attendees varies widely, we have created optional learning tracks that link related breakout sessions, thereby guiding the student with structured learning opportunities and minimizing scheduling conflicts between complimentary sessions.

Arising from our continuous quality improvement process that had just been established, we also recently introduced an optional clinical simulation exercise, supplying students with performance-based teaching and assessment. The inaugural activity in 2016 utilized standardized patients portraying a transgender woman coming to clinic with several medical and dental complaints. Teams of interprofessional students had the opportunity to employ and practice the knowledge, skills, and attitudes gained from earlier Forum activities. The structure and focus of these clinical simulations will be explored in greater depth in a subsequent manuscript.

Beyond the elective’s 10-hours of instruction, we offer networking opportunities for interested health professions students, professors, and local clinicians. An open post-Forum reception or dinner permits faculty and student interaction. Tabling by community organizations and clinical researchers throughout the Forum allows students to connect with local community resources and register for research or volunteer opportunities. Some noted benefits of networking time have included meeting previously uninvolved faculty to lead future breakout sessions, and recruiting student attendees for future Forum planning.

## Evaluation

A formal evaluation to determine the impact and effect of this unique course curriculum and structure is currently underway. Although we are unable to fully assess at this time whether the Forum goals and course objectives have been successfully met, some initial data is available and provided here.

### Participants

#### Demographics

A total of 550 individuals attended the 2015 and 2016 Forums, with 273 participants completing an online demographic survey (50% response rate) using Qualtrics (Qualtrics, Provo, USA) and 192 (35%) students formally enrolled for elective credit. The non-UCSF general public compromised 41 (12%) of the attendees. The authors lack sufficient data to accurately categorize attendees prior to 2015. Table 1 summarizes 2015–2016 participant demographics. Of these participants, 179 (66%) self-identified as female and 14 (5%) of students identified as transgender or gender nonconforming. In total, 120 (44%) of attendees identified as LGBTQI. The majority of participants identifying outside of LGBTQI communities demonstrates broad interest amongst allies and the widespread demand for curricular inclusion of LGBTQI health information.

**Table 1.** Participant demographics, 2015–2016 (*n* = 273).

Demographic	Classification	% (*n*)
Race^a^	Alaskan native	1 (3)
	Hawaiian/other Pacific Islander	1 (2)
	White	47 (127)
	Asian	38 (105)
	Black	4 (11)
	Caribbean	0 (1)
	Other	13 (35)
Hispanic, of any race		14 (37)
Previously attended forum		27 (74)
UCSF school affiliation^a^	Medicine	14 (39)
	Dentistry	9 (25)
	Pharmacy	47 (127)
	Nursing	13 (36)
	Physical therapy	4 (11)
	Grad	0 (1)
	Other	1 (2)
	Missing	12 (32)
Gender identity (regardless of transgender status)^a^	Male	31 (84)
	Female	66 (179)
	Transgender, female-to-male	0 (1)
	Transgender, male-to-female	1 (2)
	Genderqueer	3 (9)
	Other	2 (5)
Sex assigned at birth	Female	68 (185)
	Male	32 (88)
Transgender, any gender identity^b^		5 (14)
Sexual orientation	Bisexual	7 (20)
	Gay	16 (44)
	Lesbian	5 (14)
	Heterosexual	56 (153)
	Asexual	0 (1)
	Pansexual	1 (4)
	Queer	8 (21)
	Fluid	1 (3)
	Questioning	2 (5)
	Other	3 (8)

^a^ Participants are able to select multiple options.

^b^ The ‘two-step’ method was utilized for the collection of gender identity information (as opposed to a single question, as many transgender individuals will identify as both female or male *and* transgender). This practice collects both gender identity and sex assigned at birth. Transgender participants are identified as those whose birth-assigned sex and gender identity differ (e.g., male gender identity and female sex assigned at birth).

Though our curricula reached a majority non-LGBTQI audience, the demographics skewed towards heterosexual female-identified attendees, and the percentage of transgender people (5%) is over eight times the national prevalence estimate of 0.6% [9]. This suggests a need to specifically engage male and non-transgender students. The overrepresentation of LGBTQI-identified students compared to the national average likely reflects the inherent interest of LGBTQI students to learn about and address disparities within their own community. While race-concordance between patients and providers has been shown to improve patient satisfaction [9], to our knowledge no study has investigated patient-provider sexual and gender minority concordance.

The low enrollment of students for formal elective credit could be reflective of the value of the course on a student’s transcript or the elective requirement by a particular professional school. For example, in our school of medicine, there is only a 6-credit hour elective requirement and this can be satisfied by students quite rapidly and with little planning early in their curriculum; therefore, by not enrolling in course, students have the freedom to attend what sessions they feel passionate about and then depart campus to focus on other studies. The value of having this course listed on their transcript, formally printed as ‘LGBTQI Health Forum’, is minimal considering most post-graduate residency programs will not factor this into their decisions for extending an interview nor candidate rankings. On the contrary, there is a higher elective burden of 10-credit hours in the school of pharmacy and having this course listed on a pharmacy student’s transcript not only contributes to the elective requirements of the school, but signals the student’s interest in the provision of culturally competent patient care – which could impact or at least potentially influence pharmacy post-graduate training selection decisions since the demand for pharmacy residencies is higher than the supply in the USA.

#### Student intrinsic motivation and initial impact

Of students, 76% cited a reason beyond elective credit as motivation for enrollment, despite requiring weekend commitment. Students overwhelmingly indicated that their respective schools did not meet their expectations on exposure to LGBTQI health and demonstrated improved confidence in their ability to provide healthcare to LGBTQI patients upon completing the Forum. Although we cannot comment on how participant demographics have changed over time at this juncture, the authors remain encouraged by the large number of attendees each year and are committed to further analyzing the Forum’s composition moving forward.

In 2015, a formal pre- and post-forum survey was created in order to examine participant beliefs and confidence surrounding LGBTQI healthcare. Completion of the survey was voluntary and anonymous and did not affect the grade (pass/not passed) assigned to the student. The survey contained items measuring the recipient’s perception of various items and scored them on a six-point Likert-type scale (1 = strongly disagree, 6 = strongly agree), effectively eliminating the ‘neutral’ option and requiring the learner to choose a specifically defined scaled response. Responses from two separate Forums (2015 and 2016) from students that were formally enrolled in the elective were aggregated and data analysis was conducted using statistical software (Stata v.14.1, College Station, TX). Results from the surveys indicate a relative lack of LGBTQI health-related content in their core curricula, rating 2.98 on the 6-point scale believing there was adequate content (Table 2). From 2015–2016, students also reported significantly improved comfort interacting with LGBTQI patients (*p* < 0.01) following participation in the Forum. Measures of knowing where to find more information increased significantly (*p* < 0.01) as did student confidence in conducting an accurate and inclusive medical history with an LGBTQI patient (*p* < 0.01).

**Table 2.** Changes in student perspective, beliefs, and confidence related to LGBTQI health topics, pre- and post-forum, aggregated over two years.

	Pre-Forum (mean ± SD, *n* = 140)	Post-Forum (mean ± SD, *n* = 192)	Effect size: Cohen’s *d*
**Perspective**			
I believe there is adequate LGBTQI-related content in my school’s core curriculum (excluding this Forum).	2.98 ± 1.33	–	–
**Belief**			
I feel comfortable interacting with LGBTQI people.	5.26 ± 0.94	5.41 ± 0.67	0.19
I feel comfortable interacting with LGBTQI patients.*	4.81 ± 0.99	5.11 ± 0.75	0.34
I feel unprepared to provide healthcare for LGBTQI patients.*	3.39 ± 1.12	2.89 ± 1.15	0.44
**Confidence**			
I know where to access information regarding LGBTQI health issues.*	3.44 ± 1.32	4.51 ± 1.03	0.90
I can list three unique healthcare needs of LGBTQI patients.*	3.94 ± 1.50	5.13 ± 0.84	0.98
I am confident that I can conduct an accurate sexual history from LGBTQI patients.*	3.26 ± 1.41	4.54 ± 0.89	1.09
When conducting a medical history, I know questions that are uniquely relevant to LGBTQI patients.*	3.22 ± 1.42	4.67 ± 0.81	1.25

* p < 0.01 (two-tailed *t*-test); SD = standard deviation.

## Conclusion

### Reflections and next steps at our institution

The provision of LGBTQI content to health professional students is still in its infancy. This novel Forum offers an alternative model to educate students on this content within our institution’s pre-existing educational framework. Considering the known barriers of lack of provider knowledge and cultural humility, these educational efforts represent reasonable steps towards improved patient care. As with any innovative approach, however, multiple challenges remain.

First, despite having this elective course available to students for the past eight years, little data has been collected on the program. This particular course lacked sufficient documentation examining student demographics, assessment and performance, and faculty teaching in its early years. Student-organized electives, such as the one described in this paper, are often short-lived and have minimal enrollment as they are designed around a specific cohort of students interested in very specific subject areas that often preclude enrollment by the larger student body. Upon the inception of this elective, it could not have been imagined by its original organizers that the Forum would grow in acceptance and continue to exist nearly 10 years later; therefore, minimal efforts were in place initially to fully capture the rich educational data that could have existed longitudinally over the past 9 years. A more robust system to better scrutinize the curriculum and its educational impact is now in place and data continues to be actively gathered in order to better understand the potential benefits this elective and its innovative structure offers to its learners.

Second, students may repeat the elective in subsequent years for credit since we believe this encourages expansion of knowledge by attending different breakout sessions and underscores the necessity of health professional students to engage in self-directed learning. However, repeat attendance presents challenges to Forum organizers, as the plenary sessions have historically been the same topics and often employ recurrent speakers. The planning committee continues to work towards balancing the plenary course content, exposing new learners to necessary and foundational information while also challenging advanced students with more complex and controversial topics.

Lastly, we envision future Forums to leverage emerging educational technologies to enhance student participant learning and expand our audience to clinic staff and practicing clinicians through a remote learning experience. Given recent positive student feedback on new standardized patient exercises, expanded clinical simulation activities will be offered for breakout sessions. Discussions are also underway to develop a comparable program that would offer continuing education credit for licensed practitioners.

Ultimately, we hope our Forum structure serves as a model for supplying LGBTQI content into existing curricula while fostering interprofessional education. It offers an option of delivery for not only LGBTQI content, but also other underrepresented subjects without having to restructure the curriculum of an entire program. What differentiates our program from traditional elective courses is its organization by students and its multiple breakout sessions or pseudo-conference architecture allowing for individualized learning experiences and student exposure to a broader range and deeper understanding of LGBTQI health. The authors envision a time when this elective would no longer be needed and true integration of LGBTQI content into the required curriculum is a reality, but as with any major curriculum reform, smaller iterative steps are often necessary and the development of this Forum is one such initiative.

### Reflections and future directions for other institutions

We acknowledge that San Francisco’s comparatively large LGBTQI community and prominent history results in a unique availability of local patient advocates, medical and public health experts, and institutional support; however, our own institution that exists in the heart of San Francisco continues to struggle with adequate LGBTQI content delivery in all five professional schools’ curricula. Our experience suggests that successful execution ultimately relies most heavily on a core group of student organizers and a faculty champion, which likely exists within other schools, thereby opening the possibility of replicating a similar course at other universities. At our institution, a collaborating LGBT Resource Center and student participation from all five health professional schools enhances sustainability. Other institutions may look to their respective diversity offices for expertise and funding and consider professional and community organizations for additional financial support. If interprofessional opportunities within a given school are sparse, the campus may wish to collaborate with nearby external institutions. Universities with schools of public health, policy, law, or social work can expand their content exposure by integrating these disciplines into their LGBTQI curricula as well.
